# Measuring relational wellbeing: construct validity in pre-COVID-Era UK; generalizability across COVID-lockdown-Era India, Greece, and UK

**DOI:** 10.3389/fpsyg.2024.1342991

**Published:** 2024-05-17

**Authors:** Stanley O. Gaines, Pauldy Otermans, Maria Spanoudaki, Dev Aditya, Netsai Chirenda

**Affiliations:** ^1^Department of Life Sciences, Brunel University London, Uxbridge, United Kingdom; ^2^Otermans Institute, London, United Kingdom; ^3^Department of Computer Science, Brunel University London, Uxbridge, United Kingdom

**Keywords:** relational wellbeing, exploratory factor analyses, India, Greece, United Kingdom, confirmatory factor analysis, analysis of variance, COVID

## Abstract

**Aim:**

In the present studies, we examine the construct validity and criterion-related validity of a previously unpublished, eight-item measure of relational wellbeing.

**Methods:**

First, in two pre-COVID-Era pilot studies within the UK (*n*’s = 207 and 146, respectively), results of exploratory factor analyses revealed that—with the possible exception of one item regarding close relationships—the items assessed individual differences along a single dimension (i.e., relational wellbeing), rather than two distinct dimensions (i.e., social connections and close relationships). Second, in an initial pre-COVID-Era main study within the UK (*n* = 192), results of confirmatory factor analyses provided support for the hypothesized one-dimensional factor pattern, although the same problematic item from the pilot studies continued to under-perform relative to the other seven items.

**Findings:**

In a subsequent COVID-Lockdown-Era main study across India (*n* = 205), Greece (*n* = 354), and the UK (*n* = 390), results of confirmatory factor analyses established that—after omitting the same problematic item that had surfaced in the preceding studies—a one-dimensional factor pattern provided equally satisfactory fit for the three samples.

**Original value:**

Although we had not set out to test *a priori* hypotheses regarding mean similarities or differences in relational wellbeing among our COVID-Lockdown-Era studies, results of an analysis of variance revealed that persons within the UK scored significantly lower in relational wellbeing than did persons in India or Greece.

**Limitations:**

As noted above, one particular item repeatedly performed poorly in factor analyses; this item ideally should be dropped from the relational wellbeing scale in future research.

## Introduction

### Motivation

Thanks largely to the rise of the positive psychology movement during the late twentieth century and early twenty-first century, *wellbeing*—which Ryan and Deci (2001) defined as “optimal psychological functioning and experience” (p. 142)—has emerged as a major construct within the discipline of psychology (Klein and Mills, 2017). Initially throughout the Global North, and subsequently throughout the Global South, two particular forms of the wellbeing construct have gained prominence: (1) *Subjective wellbeing*, which denotes individuals’ optimal psychological *experience* (e.g., Diener et al., 2003); and (2) *psychological wellbeing*, which denotes individuals’ optimal psychological *functioning* (e.g., Ryff and Singer, 2008). Subjective wellbeing tends to be measured via a combination of (a) the Satisfaction with Life Scale (SWLS; Diener et al., 1985) and (b) the re-purposed Positive and Negative Affect Schedules (PANAS; Watson et al., 1988), the latter of which had been developed to assess individuals’ mood states (rather than individuals’ feelings about their lives, as Diener and colleagues ultimately interpreted that survey; Tov et al., 2022). Separately, psychological wellbeing tends to be measured via a survey that was described (but not published) by Ryff (1989); see also Ryff and Keyes, 1995).

### Research gap

During the same time interval that the construct of wellbeing became well-established within psychology, attempts to conceptualise and measure aspects of subjective wellbeing (e.g., Diener et al., 1985) and psychological wellbeing (e.g., Ryff, 1989) came under increasing scrutiny within the multidimensional field of development studies (White et al., 2012). In particular, the assumption that aspects of wellbeing that were operationalised originally within Global North nations could be readily generalised to Global South nations was criticised on the basis that prevailing theories and methodologies from so-called Global North nations had been invoked as normative or self-evident for all nations and had not been adequately tested within the Global North (White, 2010). Partly in response to those critiques, White et al. (2014) proposed that *inner wellbeing*—that is, individuals’ optimal psychological functioning, *even when faced with considerable adversity*—might be better-suited for understanding wellbeing among persons within the Global South than were previous Global North-driven efforts to document individual differences in wellbeing (see also Gaines, 2014; Ramirez, 2017). Some reviewers have contended that White et al.’s operationalisation of inner wellbeing helps to contextualise wellbeing as a construct (e.g., Smith and Reid, 2018).

### Research objects of the present studies

Having pioneered qualitative as well as quantitative approaches to studying inner wellbeing (e.g., White et al., 2016), White (2017) championed studies of *relational wellbeing* (i.e., individuals’ optimal psychological functioning *within the domains of social and personal relationships*, even when faced with considerable adversity) as a key aspect of inner wellbeing (Smith and Reid, 2018). In the present studies, we followed White et al.’s (2014) lead in the process of quantifying inner wellbeing in general, and relational wellbeing in particular (e.g., our adapted items that were designed to measure individuals’ inner wellbeing within the domains of *social connections* and *close relationships* are shown in Table 1; compare with domain-specific items from White et al., 2014, p. 737). We shall restrict the scope of the present paper to relational wellbeing (consistent with Spanoudaki et al., 2023, whose data comprise the present Main Study 2). One major difference between (1) the various Global South nation-specific scales by White and colleagues (e.g., Gaines, 2014, in Zambia; Ramirez, 2017, in Mexico; and White et al., 2014, in India) and (2) the present scale (s) is that we developed items for use across Global North *and* Global South contexts—a prerequisite for comparing mean scores, during COVID lockdown, in Main Study 2.

**Table 1:** Relational wellbeing items for use in the present studies.

Item	Intended domain
1. I do not feel that I really belong in this community.*	Social connections
2. If something goes wrong, I know people who can help me sort it out	Social connections
3. I feel like I have a good social life.	Social connections
4. If something happens, I am one of the last to get to know.*	Social connections
5. I have someone I can turn to if I feel stressed or low.	Close relationships
6. I often feel isolated and alone.*	Close relationships
7. I feel that there are few people in my life who really care about me.*	Close relationships
8. I have people whom I can count on, whatever happens.	Close relationships

*Reverse-worded item.

## Pilot studies: measuring relational wellbeing in pre-COVID-Era UK

At the time that we conducted the pilot studies for the present paper, we were concerned primarily with building upon the legacy that White and colleagues had built via the Wellbeing and Poverty Pathways (WPP) Project, which had advocated the assessment of inner wellbeing as one means toward understanding how individuals might be able to navigate their physical and social environments in a manner that could enable them to escape the grip of poverty (e.g., Gaines and White, 2014). In the present authors’ research within the UK, we have pursued the assessment of inner wellbeing as an end in itself; we conducted a series of pilot studies (for which results had gone unpublished prior to completion of the present paper) in which we aimed to demonstrate that White et al.’s (2014) conceptual model of inner wellbeing domains was empirically applicable to Global North contexts, just as White and colleagues had demonstrated across Global South contexts (e.g., Gaines, 2014; Ramirez, 2017). In the present subsection, we briefly summarize results of two pilot studies that are directly relevant to our overarching goal of devising inner wellbeing surveys that will yield generalizable results across sociocultural contexts; we conducted both of the pilot studies within the United Kingdom, years before the onset of COVID-19.

### Pilot Study 1

Details regarding results for Pilot Study 1 (*n* = 207) are presented in Appendix 1. With regard to the eight relational wellbeing items, non-normality statistics indicated that the distributions of scores on the items generally did not depart substantially from normality (all skewness statistics were below 2.26 in absolute value; and kurtosis statistics for all items except “I have someone I can turn to if I feel stressed or low” were below 2.26 in absolute value; see Lei and Lomax, 2005, for acceptable levels of non-normality statistics at the univariate level). Additionally, Mardia’s relative multivariate kurtosis for the relational wellbeing items as a whole was 1.34, which is acceptable regarding normality at the multivariate level (see Cain et al., 2017). Results of an exploratory factor analysis (with maximum likelihood factor extraction) using the PRELIS subroutine within LISREL 12.4.4 (Scientific Software International, 2023) technically indicated that a two-factor solution provided optimal fit to the matrix of correlations among those items. However, inspection of factor loadings revealed that the “factors” in question were *not* the conceptually distinct social connections (i.e., the domain of social relationships) and close relationships (i.e., the domain of personal relationships), but rather the methodologically distinct *positively worded items* (Factor 1, comprising three of the four positively worded items) and *negatively worded items* (Factor 2, comprising all four negatively worded items, plus—paradoxically—one of the positively worded items).

Given that such a positive–negative split historically plagued research on mental-health-adjacent constructs such as self-esteem (Carmines and Zeller, 1979), we ran the exploratory factor analysis anew using SPSS 29.0.1 (IBM, 2023), requiring a one-factor maximum-likelihood solution (indicating overall relational wellbeing) for which loadings are shown in Table 2. Also, seven of the eight items obtained loadings of 0.32 or higher, with the remaining item (i.e., “I feel that there are few people in my life who really care about me”) falling just short of that benchmark (see Tabachnick and Fidell, 2013, regarding desired cutoff levels for absolute values of factor loadings). Lastly, results of a follow-up reliability analysis indicated that the 8-item relational wellbeing scale was internally consistent for Pilot Study 1 (Cronbach’s alpha = 0.78, McDonald’s omega = 0.77).

**Table 2:** Loadings for relational wellbeing items, one-factor solution, Pilot Study 1 (*n* = 207).

Item	Factor loading
1. I do not feel that I really belong in this community.*	0.42
2. If something goes wrong, I know people who can help me sort it out.	0.66
3. I feel like I have a good social life.	0.62
4. If something happens, I am one of the last to get to know.*	0.34
5. I have someone I can turn to if I feel stressed or low.	0.74
6. I often feel isolated and alone.*	0.54
7. I feel that there are few people in my life who really care about me.*	0.28
8. I have people whom I can count on, whatever happens.	0.83

^*^Reverse-worded item. For the purposes of conducting a follow-up reliability analysis, Pilot Study 1 participants’ responses for reverse-worded items were rescored so that higher scores reflected higher levels of relational wellbeing.

### Pilot Study 2

Details concerning results for Pilot Study 2 (*n* = 146) are presented in Appendix 2. With regard to the eight relational wellbeing items, non-normality statistics indicated that the distributions of scores on the items did not depart substantially from normality (all skewness statistics were below 2.26 in absolute value; and kurtosis statistics for all items except one [i.e., the eighth item, “I have people whom I can count on, whatever happens”] were below 2.26 in absolute value). Additionally, Mardia’s relative multivariate kurtosis for the relational wellbeing items as a whole was 1.18, which is acceptable regarding normality at the multivariate level. Results of an exploratory factor analysis (with maximum likelihood factor extraction) using the PRELIS subroutine within LISREL 12.4.4 indicated that a one-factor solution offered optimal fit to the matrix of correlations among those items.

Even without requiring a one-factor solution, we found that running the exploratory factor analysis anew using SPSS 29.0.1 yielded a one-factor maximum-likelihood solution (again indicating overall relational wellbeing) for which loadings are shown in Table 3. Additionally, all eight items obtained loadings of 0.32 or higher. Finally, results of a follow-up reliability analysis indicated that the 8-item relational wellbeing scale was internally consistent for Pilot Study 2 (Cronbach’s alpha = 0.79, McDonald’s omega = 0.79).

**Table 3:** Loadings for relational wellbeing items, one-factor solution, Pilot Study 2 (*n* = 146).

Item	Factor loading
1. I do not feel that I really belong in this community.*	0.44
2. If something goes wrong, I know people who can help me sort it out.	0.49
3. I feel like I have a good social life.	0.51
4. If something happens, I am one of the last to get to know.*	0.61
5. I have someone I can turn to if I feel stressed or low.	0.71
6. I often feel isolated and alone.*	0.67
7. I feel that there are few people in my life who really care about me.*	0.51
8. I have people whom I can count on, whatever happens.	0.67

^*^Reverse-worded item. For the purposes of conducting a follow-up reliability analysis, Pilot Study 2 participants’ responses for reverse-worded items were rescored so that higher scores reflected higher levels of relational wellbeing.

## Transition to main studies: from pre-COVID-Era UK to generalizability across COVID-lockdown-Era India, Greece, and UK

Recapping the story so far, results of our pilot studies provide considerable evidence for relational wellbeing as a single construct, at least where the pre-COVID-Era UK is concerned. On the basis of results from previous studies regarding the measurement of inner wellbeing in general across the Global South nations of India (White et al., 2014), Zambia (Gaines, 2014), and Mexico (Ramirez, 2017), we did not initially anticipate that the items within the hypothesized domains of social connections and close relationships would coalesce within one underlying dimension in the Global North nation of the UK, although we note that White et al.’s (2012) critique of the literature on wellbeing was noncommittal toward the number or content of (inner) wellbeing variables. Nevertheless, long before White (2017) placed relational wellbeing in the foreground at a conceptual level, some of White’s earlier literatures reviews had alluded to the relational aspect of wellbeing as distinct from subjective and material aspects of wellbeing (e.g., White, 2010). With the possible exception of one item (i.e., “I feel that there are few people in my life who really care about me”), the eight items measuring relational wellbeing performed quite well in psychometric analyses across our pilot studies.

We acknowledge that the worldwide COVID lockdown during 2020 and 2021 may have altered individuals’ social and physical landscapes to such a degree that the factor patterns of relational wellbeing as observed among participants in our pre-COVID-Era UK pilot studies cannot be taken for granted within the COVID-Era UK, let alone other nations within the COVID-Era Global North *or* nations within the COVID-Era Global South (see Sinha, 2022) By the same token, COVID-Era scholarship on relational wellbeing as conceptualized by White (2017) has tended not to address the possibility that substantial within-person changes in the structure of relational wellbeing (or, alternatively, within-person changes in levels of relational wellbeing) may have occurred during the time interval in question (for a reflective account of such changes at the individual level, see Mukhtar, 2020). Nonetheless, our stance concerning the utility of examining the content of individual differences in relational wellbeing across sociocultural as well as sociohistorical contexts is compatible with the evolving perspective that relational wellbeing is a dynamic construct with potential for exploration from a variety of theoretical viewpoints (e.g., White and Jha, 2023).

Up to this point, the Introduction section of the present paper has been notably theory-free (notwithstanding the inherent conceptual and empirical appeal that might be associated with the construct of relational wellbeing, regardless of theoretical implications; e.g., Smith and Reid, 2018). Such lack of attention toward substantive theory generally reflects the state of the empirical literature on inner wellbeing, although some of White’s more recent writings have suggested that Ryan and Deci’s (2000) *self-determination theory* (which posits that human nature is characterized by the constructive motives of autonomy, competence, and relatedness) may be useful in a limited capacity when considering individual differences in inner wellbeing as a whole (e.g., not all persons are in an equal position to satisfy their psychological needs within a given sociocultural context; White and Jha, 2018). Although we do not deny the prospect that “universal” frameworks such as self-determination theory are viable conceptual tools for contemplating the impact of unconsciously experienced motives on individuals’ relational wellbeing, we shall turn to perspectives that address potential cultural as well as individual differences in consciously experienced cognition (following Varnum et al., 2010).

We had no *a priori* reason to expect the patterns of factor loadings for relational wellbeing items to vary across sociocultural contexts, as long as the same items are used across those contexts (an issue that is readily addressed via *confirmatory factor analyses*; see Thompson, 2004). However, *if* the patterns of factor loadings are similar across sociocultural contexts, then we will be in a position to determine whether the averaged total scores on relational wellbeing are similar or different across those contexts (see Brown, 2015, regarding the use of *multiple-group confirmatory factor analyses* when making such a determination). For example, from the vantage point of Hofstede’s (1980) *cultural dimensions theory*, one might expect persons within the ostensibly collectivistic India to score higher in relational wellbeing than would persons within the supposedly individualistic UK *and Greece* (for a review, see Matsumoto, 1996). Alternatively, from the vantage point of Triandis’s (1972) *theory of subjective culture*, the construct of collectivism encompasses family-centric as well as community-centric forms; as a result, one might expect persons within the allegedly collectivistic India *and Greece* to score higher in relational wellbeing than will persons within the reputedly individualistic UK (for a review, see Moghaddam et al., 1993).

## Goals of the main studies

In our main studies, we tested the following hypotheses concerning patterns of factor loadings among relational wellbeing items in pre-COVID-Era UK (Main Study 1) and across COVID-Lockdown-Era India, Greece, and UK (Main Study 2): (1) A one-factor model with all eight items loading significantly and positively (after re-scoring reverse-worded items) will provide satisfactory fit to the correlation data for the pre-COVID-Era UK sample (i.e., the model will not be rejected). (2) Similarly, a one-factor model with all eight items loading significantly and positively (after re-scoring reverse-worded items) will provide satisfactory fit to the correlation data for the COVID-Lockdown-Era India, Greece, and UK samples, such that the model does not differ significantly across the three samples. Additionally, we posed one research question regarding the means of total relational wellbeing scores across COVID-Lockdown-Era India, Greece, and UK (Main Study 2): How, if at all, do mean scores on relational wellbeing differ across the three nations in which we collected data? We tested Hypotheses 1 and 2 via confirmatory factor analyses (CFAs); and we sought an answer to our research question via analysis of variance (ANOVA; for further information on multivariate statistical analyses in general, see Tabachnick and Fidell, 2013).

## Main studies 1 and 2

### Materials and methods

#### Participants

##### Main Study 1: pre-COVID-Era UK sample

A total of 192 individuals (54 men, 144 women) comprised the pre-COVID-Era UK sample in Main Study 1. Regarding age, 61.1% of the pre-COVID-Era UK sample were 18–25 years of age; 27.3% were 26–40 years of age; and 11.6% were 41 years of age or older. Regarding additional demographic characteristics, 70.0% of participants were students; in relation to ethnicity, 44.9% of participants were White, 22.1% were Asian, 12.1% were Black, 11.1% were other ethnicities, and 9.1% were Mixed (an additional 0.5% did not report their ethnicity).

##### Main Study 2a: COVID-lockdown-Era India sample

A total of 205 individuals (110 men, 95 women) comprised the India sample who confirmed that they lived in India during COVID-19 lockdown. Regarding age, 58.0% of participants in the India sample were 18–25 years of age; 34.6% were 26–40 years of age; and 6.3% were 41 years of age or older (an additional 1% did not report their age). Regarding additional demographic characteristics, 51.2% were students; in relation to ethnicity, 100.0% were Asian-Indian.

##### Main Study 2b: COVID-lockdown-Era Greece sample

A total of 354 individuals (117 men, 237 women) comprised the Greece sample who confirmed that they lived in Greece during COVID-19 lockdown. Concerning age, 18.1% of participants in the Greece sample were 18–25 years of age; 38.4% were 26–40 years of age; and 43.5% were 41 years of age or older. Regarding additional demographic characteristics, 18.1% of participants indicated that they were students; in relation to ethnicity, 95.0% of participants were White, 3.3% were Mixed, 1.7% were other ethnicities, 0% were Asian, 0% were Black.

##### Main Study 2c: COVID-lockdown-Era UK sample

A total of 392 individuals (95 men, 297 women) comprised the UK sample who confirmed that they lived in the UK during COVID-19 lockdown. With regard to age, 56.4% of participants in the COVID-Lockdown-Era UK sample were 18–25 years of age; 28.1% were 26–40 years of age; and 15.6% were 41 years of age or older. Regarding additional demographic characteristics, 60.7% of participants were students; in relation to ethnicity, 61.7% of participants were White, 14.4% were Asian, 11.1% were Black, 8.3% were other ethnicities, and 4.5% were Mixed.

#### Materials

In Main Studies 1 and 2, participants completed a 28-item inner wellbeing survey that was adapted from an earlier version by White et al. (2014). The survey consisted of seven subscales (designed to measure economic confidence, agency/participation, social connections, close relationships, physical/mental health, competence/self-worth, and values/meaning as intercorrelated domains), with four items per subscale. Each item was scored according to a 5-point, Likert-type scale (1 = disagree strongly, 5 = agree strongly), with higher scores reflecting higher levels of inner wellbeing after recoding of reverse-worded items. [Although White, Gaines, and Jha did not state a rationale for including reverse-worded items in their original survey, it is clear that they were attempting to ensure that the factor structure of inner wellbeing was not confounded with method effects; see White et al., 2012 for a description of a pilot version that they abandoned precisely because such a methodological issue had arisen.]

With regard to the eight relational wellbeing items in Main Study 1, non-normality statistics (shown in Table 4) indicated that the distributions of scores on the items did not depart substantially from normality (all skewness statistics were below 2.26 in absolute value; and all kurtosis statistics likewise were below 2.26 in absolute value). Additionally, Mardia’s relative multivariate kurtosis for the relational wellbeing items as a whole was 1.14, which is acceptable regarding normality at the multivariate level. Similarly, with regard to the eight relational wellbeing items in Main Study 2, non-normality statistics (shown in Table 5) indicated that the distributions of scores on the items did not depart substantially from normality (for all three nations, skewness statistics were below 2.26 in absolute value; and for all three nations, kurtosis statistics were below 2.26 in absolute value, with the exception of the item “I have someone I can turn to if I feel stressed or low” for the Greece sample—and even that kurtosis value was exactly 2.36). Additionally, Mardia’s relative multivariate kurtosis for the relational wellbeing items as a whole was 1.18 for the India sample, 1.27 for the Greece sample, and 1.14 for the UK sample—all of which are acceptable regarding normality at the multivariate level Given that a major goal of Main Studies 1 and 2 was to determine whether a one-factor model would provide satisfactory fit to the correlational data specifically for the relational wellbeing (i.e., social connections and close relationships) items, we will report the results of reliability analyses in the Results section (after establishing the factor patterns for all of the samples in question).

**Table 4:** Non-normality statistics for relational wellbeing items, Main Study 1 (*n* = 197).

Item	Skewness	Kurtosis
1. I do not feel that I really belong in this community.*	−0.20	−0.84
2. If something goes wrong, I know people who can help me sort it out.	−0.99	0.53
3. I feel like I have a good social life.	−0.57	−0.69
4. If something happens, I am one of the last to get to know.*	−0.22	−0.56
5. I have someone I can turn to if I feel stressed or low.	−1.23	0.64
6. I often feel isolated and alone.*	0.14	−1.33
7. I feel that there are few people in my life who really care about me.*	0.47	−1.12
8. I have people whom I can count on, whatever happens.	−1.32	1.46

^*^Reverse-worded item. For the purposes of conducting a follow-up reliability analysis, Main Study 1 participants’ responses for reverse-worded items were rescored so that higher scores reflected higher levels of relational wellbeing.

**Table 5:** Non-normality statistics for relational wellbeing items, Main Study 2.

Item	Skewness	Kurtosis
*India sample (n = 205)*		
1. I do not feel that I really belong in this community.*	−0.54	−0.88
2. If something goes wrong, I know people who can help me sort it out.	−1.17	0.83
3. I feel like I have a good social life.	−0.97	0.16
4. If something happens, I am one of the last to get to know.*	−0.76	0.11
5. I have someone I can turn to if I feel stressed or low.	−0.99	−0.08
6. I often feel isolated and alone.*	−0.21	−1.26
7. I feel that there are few people in my life who really care about me.*	0.84	−0.59
8. I have people whom I can count on, whatever happens.	−1.54	1.87
*Greece sample (n = 354)*		
1. I do not feel that I really belong in this community.*	−0.34	−0.79
2. If something goes wrong, I know people who can help me sort it out.	−1.04	0.88
3. I feel like I have a good social life.	−0.72	0.03
4. If something happens, I am one of the last to get to know.*	−0.38	−0.40
5. I have someone I can turn to if I feel stressed or low.	−1.53	2.36
6. I often feel isolated and alone.*	−0.33	−1.04
7. I feel that there are few people in my life who really care about me.^*^	0.29	−1.31
8. I have people whom I can count on, whatever happens.	−1.70	2.27
*UK sample (n = 390)*		
1. I do not feel that I really belong in this community.*	−0.12	−0.70
2. If something goes wrong, I know people who can help me sort it out.	−0.97	0.52
3. I feel like I have a good social life.	−0.33	−0.91
4. If something happens, I am one of the last to get to know.*	−0.32	−0.42
5. I have someone I can turn to if I feel stressed or low.	−1.21	0.44
6. I often feel isolated and alone.*	0.08	−1.03
7. I feel that there are few people in my life who really care about me.*	0.43	−1.14
8. I have people whom I can count on, whatever happens.	−1.31	1.21

^*^Reverse-worded item. For the purposes of conducting a follow-up reliability analysis, Main Study 2 participants’ responses for reverse-worded items were rescored so that higher scores reflected higher levels of relational wellbeing.

#### Procedure

Main Studies 1 and 2 were conducted in accordance with the British Psychological Society [BPS] Code of Ethics and Conduct (BPS, 2021a) and the BPS Code of Human Research Ethics (BPS, 2021b), as were the pilot studies that we summarized in the Introduction section. First, we upheld the four ethical principles that underlie the BPS Code of Ethics and Conduct regarding the treatment of participants in general (i.e., respect; competence; responsibility; and integrity). Second, we upheld the four principles that underlie the BPS Code of Human Research Ethics concerning the treatment of human participants in particular (i.e., respect for the autonomy, privacy and dignity of individuals, groups and communities; scientific integrity; social responsibility; and maximizing benefit and minimizing harm).

Main Studies 1 and 2 were approved by the authors’ institution (for Main Study 1, pre-COVID-Era UK sample: Ref: 16599-MHR-Jun/2019-19424-3; for Main Study 2, COVID-Lockdown-Era UK and Greece samples: Ref: 16599-A-May/2020-25607-1 and COVID-Lockdown-Era India sample: Ref: 001-March2020). The relational wellbeing scale (as part of the inner wellbeing survey) was administered online using Qualtrics. Participants were recruited via social media (e.g., LinkedIn, Twitter, Facebook, WhatsApp, and Instagram); no particular pages were targeted, and the advert was shared on the authors’ personal social media accounts. For the pre-COVID-Era UK and COVID-Lockdown-Era data sets, the institution’s SONA system was used, whereby Psychology students take part in studies to receive course credits. All students from the UK attended that institution and received the relevant course credits (i.e., one credit for 15 min of participation). In terms of eligibility for the COVID-Lockdown-Era UK and Greek samples, participants had to live in one of those nations during the COVID-19 pandemic in order to take part. For the COVID-Lockdown-Era India sample, participants had to be Indian and live in India during the COVID-19 pandemic in order to be eligible. All participants had to be over 18 years old in order to take part in each of the main studies.

After opening the survey, participants in Main Studies 1 and 2 viewed a Participant Information Sheet (which provided general information about the study in question), followed by an informed consent form. The survey began with the demographic questions followed by the inner wellbeing survey (including the relational wellbeing items), as well as additional questionnaires that will not be considered further. At the end of the survey, participants were thanked for their participation and viewed a debriefing form (which explained the purpose of the research in greater detail). Each of the main studies required approximately 10 min for participants to complete. For Main Study 1, data collection took place between 18th July 2019 and 10th March 2020; for Main Study 2, data collection took place between 11th March 2020 and 29th December 2021.

## Results

### Factor pattern(s) of relational wellbeing items for Main Study 1

A matrix of zero-order correlations among relational wellbeing items for Main Study 1 (pre-COVID-Era UK sample) is presented in Table 6. After reverse-worded items were recoded, all interitem correlations were positive; and all except three correlations were significant (*p*’s < 0.05 or lower). Subsequently, the correlation matrix was entered into an *a priori* confirmatory factor analysis (CFA) with maximum likelihood solution, ridge option, and ridge constant via LISREL, specifying a 1-factor model. Unlike exploratory factor analysis (EFA), CFA yields the equivalent of *p* values for individual factor loadings, as well as goodness-of-fit statistics for the model as a whole; and CFA allows researchers to take into account uncorrelated measurement error that is associated with individual items (Thompson, 2004).

**Table 6:** Zero-order correlations among relational wellbeing items, Main Study 1 (*n* = 197).

	Correlations							
Item	1	2	3	4	5	6	7	8
1	1.00							
2	0.29	1.00						
3	0.35	0.42	1.00					
4	0.32	0.30	0.26	1.00				
5	0.33	0.52	0.36	0.17	1.00			
6	0.46	0.35	0.50	0.37	0.37	1.00		
7	0.18	0.06	0.21	0.19	0.00	0.39	1.00	
8	0.28	0.53	0.39	0.27	0.66	0.33	0.05	1.00

*Reverse-worded item. For the purposes of conducting a follow-up reliability analysis, Main Study 1 participants’ responses for reverse-worded items were rescored so that higher scores reflected higher levels of relational wellbeing. All correlations with absolute values of 0.16 or higher in magnitude were significant (*p*’s < 0.05 or lower).

1. I do not feel that I really belong in this community.*

2. If something goes wrong, I know people who can help me sort it out.

3. I feel like I have a good social life.

4. If something happens, I am one of the last to get to know.

5. I have someone I can turn to if I feel stressed or low.

6. I often feel isolated and alone.*

7. I feel that there are few people in my life who really care about me.*

8. I have people whom I can count on, whatever happens.

For Main Study 1, the 1-factor model in the *a priori* CFA provided satisfactory fit (chi-square = 16.70, *df* = 20, *NS*; root mean square error of approximation [RMSEA] = 0.00; comparative fit index [CFI] = 1.00). Furthermore, with the exception of one item, all loadings were significant and positive (*p*’s < 0.05 or lower); for the remaining item (i.e., “I feel that there are few people in my life who really care about me,” which we also flagged as slightly under-performing in Pilot Study 1), the loading was marginal and positive (*p* < 0.07). Loadings for all eight items within the 1-factor model, as produced by the *a priori* CFA for Main Study 1, are shown in Table 7.

**Table 7:** Loadings for relational wellbeing items, one-factor solution, Main Study 1 (*n* = 197).

	Factor loading	
Item	*A priori*	*A posteriori*
1. I do not feel that I really belong in this community.*	0.53	0.52
2. If something goes wrong, I know people who can help me sort it out.	0.67	0.68
3. I feel like I have a good social life.	0.63	0.61
4. If something happens, I am one of the last to get to know.*	0.44	0.42
5. I have someone I can turn to if I feel stressed or low.	0.68	0.71
6. I often feel isolated and alone.*	0.65	0.62
7. I feel that there are few people in my life who really care about me.*	0.23	0.00
8. I have people whom I can count on, whatever happens.	0.69	0.71

^*^Reverse-worded item. For the purposes of conducting a follow-up reliability analysis, Main Study 1 participants’ responses for reverse-worded items were rescored so that higher scores reflected higher levels of relational wellbeing. All factor loadings greater than 0.23 in absolute value are significant (*p*’s < 0.05 or lower).

Given that the seventh relational wellbeing item did not perform quite as well as we had expected in Main Study 1, we opted to perform a second, *a posteriori* CFA model in which the loading for the under-performing item was fixed at 0.00 (see Brown, 2015, regarding comparisons among competing CFA models for a particular sample). As was the case for the *a priori* model, the 1-factor model in the *a posteriori* CFA provided satisfactory fit (chi-square = 19.92, *df* = 21, *NS*; RMSEA = 0.00; CFI = 1.00). Moreover, the goodness-of-fit concerning the *a posteriori* model with the seventh item loading pre-set at 0.00 was only marginally worse than the goodness-of-fit regarding the *a priori* model with the seventh item allowed to vary alongside all of the other items (gain in chi-square from *a priori* to *a posteriori* models = 3.22, gain in *df* from *a priori* to *a posteriori* models = 1, *p* associated with gain in chi-square from *a priori* to *a posteriori* models <0.10). Loadings for all eight items within the 1-factor model, as produced by the *a posteriori* CFA for Main Study 1, are shown in Table 7.

In the aftermath of results for *a priori* and *a posteriori* CFAs in Main Study 1, we conducted reliability analyses on the correlation matrix among relational wellbeing items, including versus excluding the under-performing seventh item via SPSS. Results of reliability analyses indicated that, whether the seventh item was included or not, internal consistency was satisfactory for the relational wellbeing scale (including the seventh item, Cronbach’s alpha = 0.78, McDonald’s omega = 0.78; excluding the seven item, Cronbach’s alpha = 0.80, McDonald’s omega = 0.80). All things considered, we obtained clear support for our hypothesis that a 1-factor model would fit the correlational data for Main Study 1; the only departure from this overall pattern was the set of equivocal results for the seventh item.

### Factor pattern(s) of relational wellbeing items for Main study 2

#### Evaluating goodness-of-fit for *a priori* versus *a posteriori* 1-factor model(s) separately for Main Study 2a (COVID-lockdown-Era India sample)

A matrix of zero-order correlations among relational wellbeing items for Main Study 2a (COVID-Lockdown-Era India sample) is presented in Table 8. After reverse-worded items were recoded, most (but not all) correlations were positive in direction; unexpectedly, four of the correlations—all of which involved the seventh item (“I feel that there are few people in my life who really care about me,” which we also flagged as under-performing in Pilot Study 1 and Main Study 1)—were negative *after* completion of the recoding process. On the one hand, five interitem correlations were not significant or marginal; on the other hand, all four of the negative correlations that involved the seventh item were significant (i.e., *p*’s < 0.05 or lower). Subsequently, the correlation matrix was entered into an *a priori* confirmatory factor analysis (CFA) with maximum likelihood solution, ridge option, and ridge constant via LISREL, specifying a 1-factor model.

**Table 8:** Zero-order correlations among relational wellbeing items, Main Study 2.

	Correlations							
Item	1	2	3	4	5	6	7	8
*India sample (n = 205)*								
1	1.00							
2	0.07	1.00						
3	0.05	0.46	1.00					
4	0.20	0.13	0.15	1.00				
5	0.14	0.46	0.41	0.24	1.00			
6	0.16	0.06	0.11	0.30	0.17	1.00		
7	0.15	−0.16	−0.18	0.01	−0.16	0.14	1.00	
8	0.03	0.56	0.37	0.15	0.48	0.13	−0.26	1.00
*Greece sample (n = 354)*								
1	1.00							
2	0.22	1.00						
3	0.39	0.44	1.00					
4	0.20	0.13	0.21	1.00				
5	0.16	0.47	0.36	0.23	1.00			
6	0.42	0.26	0.47	0.26	0.31	1.00		
7	0.21	0.04	0.17	0.16	0.09	0.39	1.00	
8	0.25	0.43	0.34	0.21	0.52	0.28	0.02	1.00
*UK sample (n = 390)*								
1	1.00							
2	0.32	1.00						
3	0.38	0.46	1.00					
4	0.30	0.18	0.21	1.00				
5	0.24	0.52	0.35	0.20	1.00			
6	0.38	0.32	0.43	0.33	0.39	1.00		
7	0.23	0.16	0.24	0.20	0.06	0.35	1.00	
8	0.21	0.54	0.32	0.22	0.67	0.35	0.05	1.00

^*^Reverse-worded item. For the purposes of conducting a follow-up reliability analysis, Main Study 2 participants’ responses for reverse-worded items were rescored so that higher scores reflected higher levels of relational wellbeing. In Main Study 2a (India sample), all correlations with absolute values of 0.14 or higher in magnitude were significant (*p*’s < 0.05 or lower); in Main Study 2b (Greece sample) and Main Study 2c (UK sample), all correlations with absolute values of 0.13 or higher in magnitude were significant (*p*’s < 0.05 or lower).

1. I do not feel that I really belong in this community.*

2. If something goes wrong, I know people who can help me sort it out.

3. I feel like I have a good social life.

4. If something happens, I am one of the last to get to know.

5. I have someone I can turn to if I feel stressed or low.

6. I often feel isolated and alone.*

7. I feel that there are few people in my life who really care about me.*

8. I have people whom I can count on, whatever happens.

For Main Study 2a, the 1-factor model in the *a priori* CFA provided satisfactory fit (chi-square = 13.06, *df* = 20, *NS*; RMSEA = 0.00; CFI = 1.00). However, the factor pattern was decidedly mixed: Loadings for four items (“If something goes wrong, I know people who can help me sort it out,” “I feel like I have a good social life,” “If something happens, I am one of the last to get to know,” and “I have people whom I can count on, whatever happens”) were significant and positive (*p*’s < 0.05 or lower); the loading for one item (“If something happens, I am one of the last to get to know”) was significant and marginal (*p* < 0.10); loadings for two items (“I do not feel that I really belong in this community” and “I often feel isolated and alone”) were nonsignificant, albeit positive; and the loading for one item (“I feel that there are few people in my life who really care about me,” which we have already mentioned repeatedly) was significant and unexpectedly *negative* (*p* < 0.05). Loadings for all eight items within the 1-factor model, as produced by the *a priori* CFA for Main Study 2a, are shown in Table 9.

**Table 9:** Loadings for relational wellbeing items, one-factor solution, Main Study 2.

	Factor loading	
Item	*A priori*	*A posteriori*
*India sample (n = 205)*		
1. I do not feel that I really belong in this community.^*^	0.07	0.10
2. If something goes wrong, I know people who can help me sort it out.	0.63	0.63
3. I feel like I have a good social life.	0.79	0.79
4. If something happens, I am one of the last to get to know.*	0.22	0.23
5. I have someone I can turn to if I feel stressed or low.	0.58	0.58
6. I often feel isolated and alone.*	0.16	0.18
7. I feel that there are few people in my life who really care about me.*	−0.25	0.00
8. I have people whom I can count on, whatever happens.	0.29	0.90
*Greece sample (n = 354)*		
1. I do not feel that I really belong in this community.*	0.49	0.42
2. If something goes wrong, I know people who can help me sort it out.	0.60	0.62
3. I feel like I have a good social life.	0.69	0.68
4. If something happens, I am one of the last to get to know.*	0.35	0.34
5. I have someone I can turn to if I feel stressed or low.	0.61	0.63
6. I often feel isolated and alone.*	0.62	0.58
7. I feel that there are few people in my life who really care about me.*	0.26	0.00
8. I have people whom I can count on, whatever happens.	0.59	0.62
*UK sample (n = 390)*		
1. I do not feel that I really belong in this community.*	0.48	0.46
2. If something goes wrong, I know people who can help me sort it out.	0.69	0.70
3. I feel like I have a good social life.	0.60	0.58
4. If something happens, I am one of the last to get to know.*	0.37	0.36
5. I have someone I can turn to if I feel stressed or low.	0.70	0.73
6. I often feel isolated and alone.^*^	0.61	0.58
7. I feel that there are few people in my life who really care about me.^*^	0.28	0.00
8. I have people whom I can count on, whatever happens.	0.68	0.71

^*^Reverse-worded item. For the purposes of conducting a follow-up reliability analysis, Main Study 2 participants’ responses for reverse-worded items were rescored so that higher scores reflected higher levels of relational wellbeing. All factor loadings greater than 0.23 in absolute value are significant (p’s < 0.05 or lower).

Given that the seventh relational wellbeing item loaded significantly in the opposite direction to predictions in Main Study 2a, we opted to perform a second, *a posteriori* CFA model in which the loading for the unexpectedly-performing item was fixed at 0.00. As was the case for the *a priori* model, the 1-factor model in the *a posteriori* CFA provided satisfactory fit (chi-square = 16.88, *df* = 21, *NS*; RMSEA = 0.00; CFI = 1.00). In addition, the goodness-of-fit concerning the *a posteriori* model with the seventh item loading pre-set at 0.00 was only marginally worse than the goodness-of-fit regarding the *a priori* model with the seventh item allowed to vary alongside all of the other items (gain in chi-square from *a priori* to *a posteriori* models = 3.82, gain in *df* from *a priori* to *a posteriori* models = 1, *p* associated with gain in chi-square from *a priori* to *a posteriori* models <0.10). Loadings for all eight items within the 1-factor model, as produced by the *a posteriori* CFA for Main Study 2a, are shown in Table 9.

In the aftermath of results for *a priori* and *a posteriori* CFAs in Main Study 2a, we conducted reliability analyses on the correlation matrix among relational wellbeing items, including versus excluding the unexpectedly-performing seventh item via SPSS. Results of reliability analyses indicated that, when the seventh item was included, internal consistency was unsatisfactory; but when the seventh item was excluded, internal consistency was satisfactory, though slightly below the customary 0.70 cutoff (including the seventh item, Cronbach’s alpha = 0.57, McDonald’s omega could not be computed due to negative intercorrelations; excluding the seventh item, Cronbach’s alpha = 0.66, McDonald’s omega = 0.65). Taken as a whole, notwithstanding our predictions to the contrary, the factor pattern was not particularly well-defined for Main Study 2a; the seventh item proved to be most problematic but was by no means the only item to depart substantially from expectations in terms of construct validity.

#### Evaluating goodness-of-fit for *a priori* versus *a posteriori* 1-factor model(s) separately for Main Study 2b (COVID-lockdown-Era Greece sample)

A matrix of zero-order correlations among relational wellbeing items for Main Study 2b (COVID-Lockdown-Era Greece sample) is presented in Table 8. After reverse-worded items were recoded, all correlations were positive in direction; and all except three correlations—all three of which were associated with the oft-flagged seventh item (“I feel that there are few people in my life who really care about me”) were significant (*p*’s < 0.05 or lower). Subsequently, the correlation matrix was entered into an *a priori* confirmatory factor analysis (CFA) with maximum likelihood solution, ridge option, and ridge constant via LISREL, specifying a 1-factor model.

For Main Study 2b, the 1-factor model in the *a priori* CFA provided satisfactory fit (chi-square = 25.37, *df* = 20, *NS*; RMSEA = 0.00; CFI = 1.00). Additionally, all loadings were significant and positive (*p*’s < 0.05 or lower). Loadings for all eight items within the 1-factor model, as produced by the *a priori* CFA for Main Study 2b, are shown in Table 9.

In order to determine how the 1-factor model would perform if the seventh item were excluded (despite the fact that we did not encounter any problems with it in Main Study 2b), we opted to perform a second, *a posteriori* CFA model in which the loading for the item in question was fixed at 0.00. As was the case for the *a priori* model, the 1-factor model in the *a posteriori* CFA provided satisfactory fit (although the fit was marginal in this instance; chi-square = 32.23, *df* = 21, *p* < 0.06; RMSEA = 0.04; CFI = 0.92). Unlike previous comparisons that we have made so far, the goodness-of-fit concerning the *a posteriori* model with the seventh item loading pre-set at 0.00 was significantly worse than the goodness-of-fit regarding the *a priori* model with the seventh item allowed to vary alongside all of the other items (gain in chi-square from *a priori* to *a posteriori* models = 6.86, gain in *df* from *a priori* to *a posteriori* models = 1, *p* associated with gain in chi-square from *a priori* to *a posteriori* models <0.01). Loadings for all eight items within the 1-factor model, as produced by the *a posteriori* CFA for Main Study 2b, are shown in Table 9.

In the aftermath of results for *a priori* and *a posteriori* CFAs in Main Study 2b, we conducted reliability analyses on the correlation matrix among relational wellbeing items, including versus excluding the seventh item via SPSS. Results of reliability analyses indicated that, whether the seventh item was included or not, internal consistency was satisfactory for the relational wellbeing scale (including the seventh item, Cronbach’s alpha = 0.74, McDonald’s omega = 0.73; excluding the seven item, Cronbach’s alpha = 0.76, McDonald’s omega = 0.75). All in all, we obtained clear support for our hypothesis that a 1-factor model would fit the correlational data for Main Study 2b; omission of the seventh item would improve model fit significantly but would not materially affect internal consistency of the relational wellbeing scale.

#### Evaluating goodness-of-fit for *a priori* versus *a posteriori* 1-factor model(s) separately for Main Study 2c (COVID-lockdown-Era UK sample)

A matrix of zero-order correlations among relational wellbeing items for Main Study 2c (COVID-Lockdown-Era UK sample) is presented in Table 8. After reverse-worded items were recoded, all interitem correlations were positive; and all but two correlations (both of which involved the previously flagged seventh item, “I feel that there are few people in my life who really care about me”) were significant (*p*’s < 0.05 or lower). Subsequently, the correlation matrix was entered into an *a priori* confirmatory factor analysis (CFA) with maximum likelihood solution, ridge option, and ridge constant via LISREL, specifying a 1-factor model.

For Main Study 2c, the 1-factor model in the *a priori* CFA provided marginal yet satisfactory fit (chi-square = 30.86, *df* = 20, *p* < 0.06; RMSEA = 0.04; CFI = 0.92). Moreover, all loadings were significant and positive (*p*’s < 0.05 or lower). Loadings for all eight items within the 1-factor model, as produced by the *a priori* CFA for Main Study 2c, are shown in Table 9.

In order to determine how the 1-factor model would perform if the seventh item were excluded (despite the fact that we did not encounter any problems with it in Main Study 2c), we opted to perform a second, *a posteriori* CFA model in which the loading for the item in question was fixed at 0.00. Unlike the *a priori* model, the 1-factor model in the *a posteriori* CFA clearly did not provide satisfactory fit (chi-square = 39.82, *df* = 21, *p* < 0.01; RMSEA = 0.05; CFI = 0.90). Also, the goodness-of-fit concerning the *a posteriori* model with the seventh item loading pre-set at 0.00 was significantly worse than the goodness-of-fit regarding the *a priori* model with the seventh item allowed to vary alongside all of the other items (gain in chi-square from *a priori* to *a posteriori* models = 8.96, gain in *df* from *a priori* to *a posteriori* models = 1, *p* associated with gain in chi-square from *a priori* to *a posteriori* models <0.01). Loadings for all eight items within the 1-factor model, as produced by the *a posteriori* CFA for Main Study 2c, are shown in Table 9.

In the aftermath of results for *a priori* and *a posteriori* CFAs in Main Study 2c, we conducted reliability analyses on the correlation matrix among relational wellbeing items, including versus excluding the seventh item via SPSS. Results of reliability analyses indicated that, whether the seventh item was included or not, internal consistency was satisfactory for the relational wellbeing scale (including the seventh item, Cronbach’s alpha = 0.77, McDonald’s omega = 0.77; excluding the seven item, Cronbach’s alpha = 0.79, McDonald’s omega = 0.78). Overall, the results concerning construct validity were less convergent with the results concerning internal consistency for the 1-factor model as applied to the eight relational wellbeing items in Main Study 2c, compared to results for Main Study 1 (and compared to results for Main Studies 2a and 2b).

#### Evaluating goodness-of-fit for unequal-groups versus equal-groups 1-factor model(s) across Main Studies 2a, b, and c (COVID-lockdown-Era India, Greece, and UK samples)

Now that we have examined the psychometric properties of the relational wellbeing scale (adapted from the Social Connections and Close Relationships items by White et al., 2014), we are in a position to rule out retaining the seventh item (“I feel that there are few people in my life who really care about me”)—*if* we ultimately wish to proceed with an equal-groups CFA (and, depending on the outcome of that analysis, a univariate ANOVA comparing relational wellbeing scores across the three sociocultural contexts; see Tabachnick and Fidell, 2013). Therefore, we shall begin our multiple-group CFAs with a simple evaluation of goodness-of-fit concerning unequal groups—specifically, by adding up the respective chi-square values and corresponding degrees of freedom for the *a posteriori* models that we have already reported in the present subsection (see Brown, 2015). A total chi-square of 88.93 (16.88 + 32.23 + 39.82), which is associated with a total *df* of 63, indicates that an “unequal-groups” model (whereby no parameter is constrained to be identical across nations) is significant (*p* < 0.05), indicating that such a model does not yield satisfactory fit to the cross-nation correlational data.

By way of contrast, we entered the interitem correlation matrices for the COVID-Lockdown-Era samples from India, Greece, and the UK into an equal-groups, 1-factor CFA whereby all fixed and freed parameters were constrained to be identical across sociocultural contexts. Results indicated that—unlike the unequal-groups model—the equal-groups model provided satisfactory fit to the data (chi-square = 108.21, *df* = 93, *NS*; RMSEA = 0.02; CFI = 0.97). Furthermore, the goodness-of-fit concerning the equal-groups model was not significantly or marginally worse than the goodness-of-fit regarding the unequal-groups model (gain in chi-square = 19.28; gain in corresponding *df* = 30; *p* associated with gain in chi-square from unequal-groups to equal-groups model *NS*). Given that we found it necessary to omit Item 7 from the equal-groups model, we concluded that results of the final CFA mostly (but not fully) supported our second hypothesis concerning the pattern of factor loadings. A matrix of factor loadings summarizing across the three nations is shown in Table 10.

**Table 10:** Loadings for relational wellbeing items, equal-groups one-factor solution, Main Study 2 (combined *n* = 949).

Item	Factor loading
1. I do not feel that I really belong in this community.*	0.39
2. If something goes wrong, I know people who can help me sort it out.	0.67
3. I feel like I have a good social life.	0.64
4. If something happens, I am one of the last to get to know.*	0.34
5. I have someone I can turn to if I feel stressed or low.	0.69
6. I often feel isolated and alone.*	0.49
7. I feel that there are few people in my life who really care about me.*	0.00
8. I have people whom I can count on, whatever happens.	0.71

^*^Reverse-worded item.

Finally, having shown that an equal-groups 1-factor model (omitting Item 7) yielded satisfactory fit (even as an unequal-groups 1-factor model failed to yield satisfactory fit), we conducted a univariate analysis of variance (ANOVA) with sociocultural context (India versus Greece versus the UK) as a predictor of individuals’ relational wellbeing. Means and standard deviations for relational wellbeing across the three COVID-Lockdown-Era samples are presented in Table 11. Results of the ANOVA indicated that, in general, sociocultural context was a significant predictor of relational wellbeing (*F* [2, 946 *df*] = 20.86, *p* < 0.01). Additionally, results of *post hoc* Scheffe tests revealed that (1) persons in the UK scored significantly lower on relational wellbeing than did persons in India or Greece; whereas (2) persons in India and Greece did not score significantly or marginally different from each other on relational wellbeing. Therefore, results of the ANOVA provided a definitive answer to our research question: Sociocultural context *did* matter to relational wellbeing, but not in terms of a straightforward Global South advantage over the Global North.

**Table 11:** Means and standard deviations for relational wellbeing scores among persons in COVID-lockdown-Era India, Greece, and the UK (Main Studies 2a, b, and c).

Nation	N	M	SD
India	205	26.86	4.71
Greece	354	26.73	4.74
UK	390	24.66	5.31

Before proceeding further, we briefly note results of a univariate analysis of covariance (ANCOVA), in which we added participants’ age as a covariate of relational wellbeing. On the one hand, age emerged as a significant positive predictor of relational wellbeing (beta weight = 0.45, *t* = 2.10, *p* < 0.05). On the other hand, not only did sociocultural context remain significant as a predictor of relational wellbeing; but all of the aforementioned nation-by-nation similarities and differences in means remained unchanged.

## Discussion

For the most part, results of Main Studies 1 and 2 supported our hypotheses regarding factor patterns of relational wellbeing, before and during the COVID Lockdown Era. If we add results from Pilot Studies 1 and 2, then the picture for the UK becomes that much clearer: Keeping in mind one problematic item throughout most of the samples, the psychometric properties of our scale affirm the construct validity of a one-factor model, rather than a two-factor (i.e., Social Connections and Close Relationships) model. Also, when we expanded the scope of our research to include samples from India and Greece alongside UK during COVID lockdown, the need to set aside the problematic item in question became even more pronounced. Lastly, although we were interested primarily in generalizability of factor patterns (instead of mean differences across sociocultural contexts), we were pleasantly surprised to obtain intriguing differences and similarities, depending upon specific Global North-Global South (or Global North-Global North) comparisons. All in all, neither the incidence of COVID lockdown nor the expansion of our programmatic research across multiple sociocultural contexts *per se* appeared to be relevant to the factor patterns that we observed.

Why did sociocultural context influence individuals’ relational wellbeing in the specific ways that we were able to document? In the Introduction section of the present paper, we briefly alluded to Triandis’s (1972) theory of subjective culture as a potential source of insight regarding sociocultural differences and similarities in relational wellbeing: To the extent that individuals embrace an interdependent social orientation as promoted via collectivistic values within a given society, those individuals presumably will experience high levels of relational wellbeing (see also Varnum et al., 2010). It turns out that some of Triandis’s own writings associated an interdependent social orientation with persons in the Global East, which can help explain why persons in India generally scored higher in relational wellbeing, compared to persons in the Global West nation of the UK (e.g., Triandis, 1999). However, White has suggested that an interdependent social orientation as conceptualized within Triandis’s theory can be associated as readily with persons in the Global South, which *might* help explain why persons in Greece (literally between North and South) generally scored higher in relational wellbeing, compared to persons in the Global North nation of the UK (e.g., White and Jha, 2023).

Lastly, why did the item “I feel that there are few people in my life who really care about me” prove to be so problematic throughout our pre-COVID-Era *and* COVID-Lockdown-Era samples? Perhaps the most analogous item that one can find within White et al.’s (2014) Time 2 inner wellbeing survey within India is the question “How much do people in your house care for you?” (p. 737). As it happens, levels of non-normality statistics (i.e., skewness as well as kurtosis) were elevated for White, Gaines, and Jha’s item; although non-normality was a non-issue for our version of the item, it is possible that our process of taking the item literally out of its sociostructural (i.e., household) context resulted in an item that no longer possessed adequate construct validity for a scale that was intended to measure relational wellbeing (for a discussion regarding the limits of skewness and kurtosis statistics as diagnostic indicators of construct validity or lack thereof, see Cain et al., 2017). Fortunately for our purposes, no other item within the relational wellbeing scale posed such persistent difficulties on psychometric grounds within any of our samples (for a discussion concerning psychometric characteristics of predecessors to the present items, see White et al., 2014).

### Strengths, limitations, and future directions

Certain strengths characterize the present studies. For instance, our use of quantitative methods to conduct large-scale studies of individual differences in relational wellbeing across three large pre-COVID-Era samples (all within the UK) and three large COVID-Lockdown-Era samples (covering India, Greece, and the UK) complements White’s use of qualitative methods to revisit case-study data from one or more individuals concerning relational wellbeing in one pre-COVID-Era sample (from the Global South nation of Zambia; e.g., White and Jha, 2020). Moreover, our comparisons of mean scores on relational wellbeing across COVID-Lockdown-Era samples in India, Greece, and the UK enabled us to rule out Hofstede’s (1980) cultural dimensions theory as an explanatory framework, even as we retained Triandis’s (1972) theory of subjective culture (see also White and Jha, 2023). Lastly, the fact that we were able to replicate the factor pattern of loadings for relational wellbeing items (setting aside the single problematic item) across so many societal and temporal contexts would seem to bode well for the construct of relational wellbeing as a viable complement to life satisfaction, positive affect, and negative affect as constructs of subjective wellbeing (see also White, 2017).

By the same token, certain shortcomings characterize the present studies. For example, we were able to collect large-scale quantitative data on relational wellbeing from multiple nations during COVID lockdown at a single point in time; ideally, we would have been able to collect data from those nations on two or more occasions during lockdown, in order to conduct exploratory as well as confirmatory factor analyses on data from separate samples within each nation (see Thompson, 2004). Furthermore, although we interpreted our results concerning sociocultural group differences and similarities in relational wellbeing from the perspective of Triandis’s (1972) theory of subjective culture, one might argue that Markus and Kitayama’s (1991) *self-construal theory* as elaborated by Cross and Madson (1997) to distinguish among independent, collective-interdependent, and relational-interdependent mental representations of self and others would be equally well-suited to explain our results (see Varnum et al., 2010). Finally, one might reasonably ask whether the construct of relational wellbeing in the present studies is subsumed by the dimension of *positive relations with others* that, in turn, forms part of the broader construct of psychological wellbeing (which had been cited approvingly by White et al., 2012).

Future researchers might wish to pursue large-scale quantitative research on individual differences in relational wellbeing across multiple nations in the post-COVID Lockdown Era, with regard to factor patterns as well as mean similarities and differences (see Brown, 2015). Also, future researchers may want to examine conceptual and empirical links between (a) the individual-difference variable of relational wellbeing, as operationalized in the present studies; and (b) the similarly named couple-difference variable of *relationship well-being*, as operationalized by relationship scientists (see Gaines, 2023). In addition, future researchers may find it useful to investigate conceptual and empirical links between relational wellbeing and culturally relevant individual-difference variables such as cultural values and self-construals (see Triandis, 1995). Lastly, future researchers might be curious about conceptual and empirical links between relational wellbeing and aspects of psychological wellbeing—not just because of the possible overlap between relational wellbeing and positive relations with others, but also because the two sets of constructs seem to be at least as compatible as subjective wellbeing and psychological wellbeing dimensions (Gaines, 2020).

## Conclusion

At the beginning of the present paper, we alluded to the importance of wellbeing as a defining construct within the Global North-led positive psychology movement during the early twenty-first century (see also Seligman and Csikszentmihalyi, 2000). In turn, the Global North-championed construct of life satisfaction has become nearly synonymous with wellbeing among positive psychologists (most notably Diener, 2000). The present paper is by no means the first to suggest that alternative, Global South-promoted constructs such as relational wellbeing ought to receive comparable attention within positive psychology (for an early example, see White et al., 2012). Nonetheless, to our knowledge, the series of studies that we have reported in this paper offer the most extensive evidence to date that relational wellbeing simultaneously stands as a scientifically valid concept (as scholars in positive psychology would be within their rights to expect, in our view) *and* a truly cross-cultural concept (as scholars in development studies would be within their rights to expect, in our opinion; see also Smith and Reid, 2018). We hope that our success in repeatedly providing empirical support for construct validity of our relational wellbeing scale, before and during the COVID Era, will help inspire follow-up studies across the globe.

## Data availability statement

The raw data supporting the conclusions of this article will be made available by the authors, without undue reservation.

## Ethics statement

All of the studies involved humans and were approved by Brunel University London, United Kingdom (for Pilot Study 1, UK Sample: Ref.: 3365-LR-Jul/2016- 3384-1; for Pilot Study 2, UK Sample: Ref: 10328-LR-Mar/2018-12043-2; for Main Study 1, pre-COVID-Era UK sample: Ref: 16599-MHR-Jun/2019-19424-3; for Main Study 2, COVID-Lockdown-Era UK and Greece samples: Ref: 16599-A-May/2020-25607-1 and COVID-Lockdown-Era India sample: Ref: 001-March2020). The studies were conducted in accordance with the local legislation and institutional requirements. The participants provided their written informed consent to participate in these studies.

## Author contributions

SG: Conceptualization, Data curation, Formal analysis, Methodology, Project administration, Writing – original draft. PO: Conceptualization, Data curation, Formal analysis, Investigation, Methodology, Project administration, Writing – review & editing. MS: Conceptualization, Data curation, Formal analysis, Investigation, Methodology, Project administration, Writing – review & editing. DA: Conceptualization, Data curation, Investigation, Methodology, Project administration, Writing – review & editing. NC: Data curation, Formal analysis, Investigation, Methodology, Project administration, Validation, Writing – review & editing.
