# Supplementary material for: Measuring relational wellbeing: construct validity in pre-COVID-Era UK; generalizability across COVID-lockdown-Era India, Greece, and UK
Source: Front Psychol. 2024 May 17;15:1342991. doi: 10.3389/fpsyg.2024.1342991 (PMC11157432; doi:10.3389/fpsyg.2024.1342991)
Supplement: Supplementary file 1 [file Data_Sheet_1.PDF]

APPENDIX 1:

ADDITIONAL DESCIRPTIVE STATISTICS

FOR PILOT STUDY 1

Table 1.1:

Demographic Statistics for Pilot Study 1 (*n* = 207)

Gender		
Category	*N*	*Percentage*
Male	54	26.1
Female	151	72.9
Prefer not to say	0	0.0
[Missing]	2	1.0

Age (years)		
Category	*N*	*Percentage*
18-25	71	34.3
26-40	84	40.6
41 and older	41	19.8
[Missing]	11	5.3

Ethnic group membership		
Category	*N*	*Percentage*
White	72	34.8
Asian	80	38.6
Black	42	20.3
Other	5	2.4
Mixed	8	3.9
[Missing]	0	0.0

Table 1.2:

Non-Normality Statistics for Pilot Study 1 (*n* = 207)[1]

Item	*Skewness*	*Kurtosis*
1. I don't feel that I really belong in this community.*	-.53	-.42
2. If something goes wrong, I know people who can help me sort it out.	-.97	.68
3. I feel like I have a good social life.	-.61	-.34
4. If something happens, I am one of the last to get to know.*	-.52	.40
5. I have someone I can turn to if I feel stressed or low.	-1.75	2.89
6. I often feel isolated and alone.*	-.64	-.29
7. I feel that there are few people in my life who really care about me.*	-.44	-1.22
8. I have people whom I can count on, whatever happens.	-1.54	1.83

[1]NOTE: *Reverse-worded item. For the purposes of conducting a follow-up reliability analysis, Pilot Study 1 participants' responses for reverse-worded items were rescored so that higher scores reflected higher levels of relational wellbeing.

Table 1.3:

Zero-Order Correlations among Relational Wellbeing Items, Pilot Study 1 (*n* = 207)[2]

Correlations

Item	*1*	*2*	*3*	*4*	*5*	*6*	*7*	*8*
1	1.00							
2	.25	1.00						
3	.42	.39	1.00					
4	.26	.14	.32	1.00				
5	.20	.54	.42	.20	1.00			
6	.36	.29	.46	.36	.29	1.00		
7	.26	.06	.22	.21	.17	.30	1.00	
8	.29	.56	.46	.21	.67	.44	.21	1.00

[2]NOTE: *Reverse-worded item. For the purposes of conducting a follow-up reliability analysis, Pilot Study 1 participants' responses for reverse-worded items were rescored so that higher scores reflected higher levels of relational wellbeing. All correlations with absolute values of .14 or higher in magnitude were significant (*p*'s < .05 or lower).

1. I don't feel that I really belong in this community.*
2. If something goes wrong, I know people who can help me sort it out.
3. I feel like I have a good social life.
4. If something happens, I am one of the last to get to know.
5. I have someone I can turn to if I feel stressed or low.
6. I often feel isolated and alone.*
7. I feel that there are few people in my life who really care about me.*
8. I have people whom I can count on, whatever happens.

Table 1.4:

Communalities for Relational Wellbeing Items, Original 2-Factor Solution, Pilot Study 1 (*n* = 207)[3]

Item	*Communality* Initial	*Communality* Extraction
1. I don't feel that I really belong in this community.*	.24	.17
2. If something goes wrong, I know people who can help me sort it out.	.40	.44
3. I feel like I have a good social life.	.39	.39
4. If something happens, I am one of the last to get to know.*	.18	.11
5. I have someone I can turn to if I feel stressed or low.	.50	.55
6. I often feel isolated and alone.*	.53	.30
7. I feel that there are few people in my life who really care about me.*	.14	.08
8. I have people whom I can count on, whatever happens.	.56	.68

[3]NOTE: *Reverse-worded item. For the purposes of conducting a follow-up reliability analysis, Pilot Study 1 participants' responses for reverse-worded items were rescored so that higher scores reflected higher levels of relational wellbeing.

Table 1.5:

Loadings for Relational Wellbeing Items, Original 2-Factor Solution, Pilot Study 1 (*n* = 207)[4]

	Factor loading	
Item	*Positive*	*Negative*
9. I don't feel that I really belong in this community.*	-.03	.59
10. If something goes wrong, I know people who can help me sort it out.	.71	-.03
11. I feel like I have a good social life.	.24	.52
12. If something happens, I am one of the last to get to know.*	-.08	.54
13. I have someone I can turn to if I feel stressed or low.	.86	-.10
14. I often feel isolated and alone.*	.05	.66
15. I feel that there are few people in my life who really care about me.*	-.07	.45
16. I have people whom I can count on, whatever happens.	.80	.06

[4]NOTE: *Reverse-worded item. For the purposes of conducting a follow-up reliability analysis, Pilot Study 1 participants' responses for reverse-worded items were rescored so that higher scores reflected higher levels of relational wellbeing.
