# Supplementary material for: Measuring relational wellbeing: construct validity in pre-COVID-Era UK; generalizability across COVID-lockdown-Era India, Greece, and UK
Source: Front Psychol. 2024 May 17;15:1342991. doi: 10.3389/fpsyg.2024.1342991 (PMC11157432; doi:10.3389/fpsyg.2024.1342991)
Supplement: Supplementary file 2 [file Data_Sheet_2.PDF]

APPENDIX 2:

ADDITIONAL DESCIRPTIVE STATISTICS

FOR PILOT STUDY 2

Table 2.1:

Demographic Statistics for Pilot Study 2 (initial *n* = 177)

Gender		
Category	*N*	*Percentage*
Male	60	33.9
Female	108	61.0
Prefer not to say	1	0.6
[Missing]	8	4.5

Age (years)		
Category	*N*	*Percentage*
18-25	60	33.9
26-40	82	46.3
41 and older	28	15.8
[Missing]	7	4.0

Ethnic group membership		
Category	*N*	*Percentage*
White	102	57.6
Asian	48	27.1
Black	9	5.1
Other	7	4.0
Mixed	6	3.4
[Missing]	5	2.8

Table 2.2:

Non-Normality Statistics for Pilot Study 2 (final *n* = 146)[1]

Item	*Skewness*	*Kurtosis*
1. I don't feel that I really belong in this community.*	-.58	-.51
2. If something goes wrong, I know people who can help me sort it out.	-1.05	1.63
3. I feel like I have a good social life.	-.69	-.33
4. If something happens, I am one of the last to get to know.*	-.37	-.49
5. I have someone I can turn to if I feel stressed or low.	-1.37	1.21
6. I often feel isolated and alone.*	-.34	-1.20
7. I feel that there are few people in my life who really care about me.*	-.29	-1.41
8. I have people whom I can count on, whatever happens.	-1.63	2.71

[1]NOTE: *Reverse-worded item. For the purposes of conducting a follow-up reliability analysis, Pilot Study 2 participants' responses for reverse-worded items were rescored so that higher scores reflected higher levels of relational wellbeing.

Table 2.3:

Zero-Order Correlations among Relational Wellbeing Items, Pilot Study 2 (final *n* = 146)[2]

	Correlations							
Item	*1*	*2*	*3*	*4*	*5*	*6*	*7*	*8*
1	1.00							
2	.26	1.00						
3	.35	.28	1.00					
4	.27	.24	.24	1.00				
5	.26	.34	.31	.43	1.00			
6	.35	.27	.41	.52	.41	1.00		
7	.30	.23	.23	.35	.34	.37	1.00	
8	.19	.41	.33	.35	.61	.37	.28	1.00

[2]NOTE: *Reverse-worded item. For the purposes of conducting a follow-up reliability analysis, Pilot Study 2 participants' responses for reverse-worded items were rescored so that higher scores reflected higher levels of relational wellbeing. All correlations with absolute values of .19 or higher in magnitude were significant (p's < .05 or lower).

1. I don't feel that I really belong in this community.*
2. If something goes wrong, I know people who can help me sort it out.
3. I feel like I have a good social life.
4. If something happens, I am one of the last to get to know.
5. I have someone I can turn to if I feel stressed or low.
6. I often feel isolated and alone.*
7. I feel that there are few people in my life who really care about me.*
8. I have people whom I can count on, whatever happens.

Table 2.4:

Communalities for Relational Wellbeing Items, Pilot Study 2,

Initial/Final 1-Factor Solution (final *n* = 146)[3]

Item	*Communality* Initial	*Communality* Extraction
1. I don't feel that I really belong in this community.*	.22	.20
2. If something goes wrong, I know people who can help me sort it out.	.22	.24
3. I feel like I have a good social life.	.26	.26
4. If something happens, I am one of the last to get to know.*	.35	.38
5. I have someone I can turn to if I feel stressed or low.	.45	.50
6. I often feel isolated and alone.*	.41	.44
7. I feel that there are few people in my life who really care about me.*	.22	.26
8. I have people whom I can count on, whatever happens.	.44	.44

[3]NOTE: *Reverse-worded item. For the purposes of conducting a follow-up reliability analysis, Pilot Study 2 participants' responses for reverse-worded items were rescored so that higher scores reflected higher levels of relational wellbeing.
